# Autoantibodies targeting to GPER1 promote monocyte cytokines production and inflammation in systemic lupus erythematosus

**DOI:** 10.1038/s41392-022-01294-3

**Published:** 2023-03-03

**Authors:** Xinwei Zhang, Hongyan Qian, Yangchun Chen, Yuanhui Wu, Yuechi Sun, Yan He, Shiju Chen, Guixiu Shi, Yuan Liu

**Affiliations:** 1 Department of Rheumatology and Clinical Immunology, the First Affiliated Hospital of Xiamen University, School of Medicine, Xiamen University, Xiamen, 361000 China; 2 Xiamen Municipal Clinical Research Center for Immune Diseases, Xiamen, 361000 China; 3 Xiamen Key Laboratory of Rheumatology and Clinical Immunology, Xiamen, 361000 China

**Keywords:** Rheumatic diseases, Immunology

**Dear Editor**,

Systemic lupus erythematosus (SLE), one of the most common autoimmune diseases in reproductive females, is a multifactorial disease involving genetic, environmental, and hormonal factors.^1^ It occurs at a 9:1 female-to-male ratio, and the sex predisposition suggests that the estrogen system plays an essential role in the developing of SLE. The effects of estrogen have traditionally been attributed to the classical nuclear estrogen receptors (ERs), ERα, and ERβ, which predominantly regulate transcription. Guanine nucleotide-binding protein-coupled estrogen receptor 1 (GPER1) is a transmembrane receptor for estrogen that mediates several rapid cellular effects of estrogen.^2^ Large amounts of autoantibodies characterize SLE, and anti-estrogen receptor autoantibodies are likely to affect the function of immune cells in SLE but are not yet fully understood. A high titer of anti-ERα autoantibodies has been identified in patients with SLE and was significantly correlated with disease activity, whereas anti-ERβ antibodies were not present in any of the patients with SLE.^3^ In contrast, the presence of anti-GPER1 autoantibodies in patients with SLE remains unstudied. Therefore, this study aimed to identify the pathogenic roles of autoantibodies to GPER1 in SLE.

Serum IgG immunoreactivity to GPER1 in 117 patients with SLE was detected using ELISA. We found that the SLE group’s OD values were significantly higher than those of other autoimmune diseases and healthy donors (Fig. 1a). Autoantibodies to GPER1 were detected in 39.3% (46/117) of patients with SLE. In contrast, these autoantibodies were identified in only 3.5% (2/56) of patients with RA, 8.3% (5/60) of patients with SS, 6.6% (4/61) of patients with AS, 9.1% (6/66) of patients with gout, and 5.7% (4/70) of healthy controls. Additionally, the rate of anti-GPER1 IgG-positivity in patients with SLE was significantly higher (Supplementary Table S1). The representative anti-GPER1 IgG-positive serum samples in SLE and healthy controls were further confirmed by Western blotting (Fig. 1b).

**Fig. 1:**
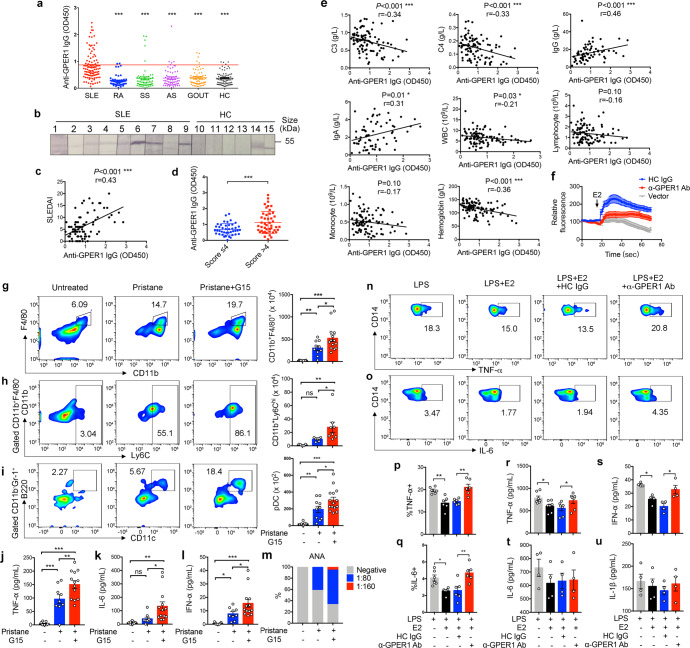
Autoantibodies to GPER1 promote monocyte cytokines production by blocking GPER1 signaling and are associated with disease activity in SLE. **a** The titer of anti-GPER1 IgG in patients with SLE (*n* = 117), rheumatoid arthritis (RA) (*n* = 56), Sjogren’s syndrome (SS) (*n* = 60), ankylosing spondylitis (AS) (*n* = 61), Gout (*n* = 66), and healthy controls (*n* = 70) was measured by ELISA. The cutoff value was defined as the mean OD value of the healthy controls+2 SDs. One-way ANOVA was used to analyze data for differences. Asterisks represent statistically significant differences from the SLE group. **b** Represented serum samples from patients with SLE and healthy controls were further confirmed by western blotting. The serum samples comprised the 10 patients with the highest OD values and the 5 randomly selected healthy controls. **c** Correlation between SLEDAI score and titer of anti-GPER1 IgG in 86 patients with SLE. Data were analyzed using Spearman’s correlation coefficients. **d** The titer of anti-GPER1 IgG in patients with SLEDAI scores of >4 and patients with SLEDAI scores of ≤4. Unpaired t-test was used to analyze data for differences. **e** Correlation between serum anti-GPER1 autoantibodies and clinical parameters in patients with SLE. Data were analyzed using Spearman’s correlation coefficients. **f** 10 μg/mL α-GPER1 Abs or HC IgG were added to GPER1-transfected HEK293 cells for 1 h and then loaded with Fluo-4 AM. Cells were stimulated by 100 nM 17β-estradiol and constantly observed for 70 s. Vector-transfected cells were used as a control. The fluorescence intensity of cells before and after the stimulation was obtained by confocal microscopy and evaluated by Leica System Analysis Software. **g**–**i** C57BL/6 mice were treated with or without pristane. After 2 days, mice were i.p. injected with G15 or DMSO as control every 2 days for 4 weeks. Subpopulations of peritoneal cells were identified by flow cytometry as macrophages (CD11b,^+^ F4/80^+^), monocytes (CD11b^+^, F4/80^−^, Ly6C^high)^, and pDCs (CD11b^−^, Gr-1^+^, CD11c^+^, B220^+^). Shown is the total number of each population. One-way ANOVA was used to analyze data for differences (*n* = 5–15). **j**–**l** TNF-α, IL-6, and IFN-α production in peritoneal lavage fluid was detected by ELISA (*n* = 9–13). One-way ANOVA was used to analyze data for differences. **m** Detection of ANA using HEP-2 slides. Sera were tested for IgG-ANA (*n* = 9–18). **n**–**q** PBMCs were stimulated with LPS, LPS + E2, LPS + E2 + α-GPER1 Abs, or LPS + E2 + HC IgG for 4 h. Intracellular staining of TNF-α and IL-6 in CD14^+^ monocytes (*n* = 6). One-way ANOVA was used to analyze data for differences. **r**–**u** Monocytes were isolated from PBMCs by magnetic cell sorting and were stimulated with α-GPER1 Abs with the presence of LPS and E2 for 72 h. The inflammatory cytokines in the supernatant were measured by ELISA (*n* = 4–7). Data are represented as mean ± SEM. One-way ANOVA was used to analyze data for differences. Data are representative of 3 independent experiments. *P < 0.05, **P < 0.01, ***P < 0.001

Among 117 patients with SLE, 86 patients had complete clinical data and were evaluated for disease activity with SLEDAI scores. Spearman’s rank analysis showed that the OD value of anti-GPER1 IgG in the sera of patients with SLE positively correlated with the SLEDAI scores (Fig. 1c). We found that patients with higher disease activity (SLEDAI > 4) had a higher anti-GPER1 IgG level than those with lower disease activity (SLEDAI ≤ 4) (Fig. 1d). Additionally, the differences in clinical characteristics in the anti-GPER1 IgG-positive and anti-GPER1 IgG-negative patients with SLE were analyzed. A higher prevalence of serositis was observed in the anti-GPER1 IgG-positive patients than in anti-GPER1 IgG-negative patients (Supplementary Table S2). Furthermore, Spearman’s rank analysis showed that the level of anti-GPER1 IgG was positively related to IgG and IgA, and negatively correlated with levels of complement 3, complement 4, and hemoglobin level (Fig. 1e).

Functional antibodies targeting GPCRs can trigger or block intracellular signaling pathways, resulting in agonistic or antagonistic effects respectively. We purified anti-GPER1 antibodies (α-GPER1 Abs) from sera of patients with SLE for in vitro experiments to confirm whether it is functional antibodies that can activate or inhibit intracellular GPER1 signaling pathways. Using 17β-estradiol (E2) as stimuli, GPER1 was activated resulting in increased cAMP production, intracellular Ca,^2+^ and ERK1/2 phosphorylation. To exclude the effect of classical ERs, HEK293 cells, which do not express ERα or ERβ, were transfected with GPER1 and incubated with α-GPER1 Abs or antibodies isolated from healthy controls (HC Abs) for 1 h before being loaded with Ca^2+^ indicator Fluo-4 AM. Whereas E2 stimulation of HC IgG-pretreated cells resulted in the mobilization of intracellular calcium, preincubation of the cells with α-GPER1 Abs essentially abolished the Ca^2+^ influx induced by E2 (Fig. 1f), supporting the blocking effect of anti-GPER1 autoantibodies on GPER1-mediated estrogen signaling.

To simulate in vivo function of anti-GPER1 autoantibodies, we injected G15, a specific inhibitor of GPER1,^4^ into pristane-induced SLE mice every 2 days for 4 weeks. Intraperitoneal injection of pristane induced immune cell infiltration in abdominal cavities, and the peritoneal lavage fluid was collected for cell counts and assessments of cytokine levels. The Numbers of infiltrating macrophages, plasmacytoid dendritic cells (pDCs), and Ly6C^high^ monocytes in the G15 treatment group were significantly increased in contrast to the control, while numbers of T cells and B cells were comparable (Fig. 1g–i, Supplementary Fig. S1a–c). We also observed increased IL-6, TNF-α, and IFN-α in peritoneal lavages from the G15 treatment group compared to the control group (Fig. 1j–l). Moreover, G15 treatment increased the titer of serum ANA in SLE mice (Fig. 1m). Therefore, the results above suggested that inhibition of GPER1 exacerbated inflammation in pristane-induced SLE mice.

As described above, inhibition of GPER1 promoted macrophages, pDCs, and Ly6C^high^ monocytes infiltration and inflammatory cytokines secretion. Therefore, we speculated that anti-GPER1 autoantibodies might act as GPER1-blocking antibodies to regulate the function of myeloid immune cells. We assessed the expression of GPER1 in PBMC subsets and found that monocytes expressed a higher level of GPER1 compared to T cells, NK cells, DCs, and NKT cells, whereas B cells expressed a comparable level of GPER1 on the cell surface with monocytes (Supplementary Fig. S2a).

To further confirm whether anti-GPER1 autoantibodies affect cytokine secretion of monocyte, PBMCs of healthy donors were treated with LPS, LPS + E2, LPS + E2 + α-GPER1 Abs, or LPS + E2 + HC IgG for 4 h. Flow cytometric staining was performed for surface CD14 to gate monocytes, followed by intracellular staining for TNF-α and IL-6 (Fig. 1n–q). We found that LPS-induced production of TNF-α and IL-6 in CD14^+^ monocyte was inhibited by E2, whereas treatment with α-GPER1 Abs recovered these levels. Additionally, monocytes isolated from PBMC were treated with α-GPER1 Abs in the presence of LPS and E2 for 72 h, and the supernatants were collected to measure the inflammatory cytokines. Levels of TNF-α and IFN-α were lower with the presence of E2 and increased considerably after incubation with α-GPER1 Abs, whereas levels of IL-6 and IL-1β were comparable between each group (Fig. 1r–u). These results indicated that α-GPER1 Abs reversed the production of TNF-α and IFN-α in monocytes by abrogating the anti-inflammatory capacity of E2. Treatment with α-GPER1 Abs did not affect CD80, CD86, HLA-DR, and TLR4 expression and the survival of monocytes (Supplementary Fig. S3a–e).

SLE is an autoimmune disease characterized by producing of a wide variety of autoantibodies. We reported the presence of anti-GPER1 autoantibodies in patients with SLE for the first time. Notably, a significant association between anti-GPER1 IgG level and disease activity was found, implying that these autoantibodies could be used as potential serological biomarkers for SLE diagnosis and activity evaluation. Analysis of the effects of α-GPER1 Abs indicated that they acted as blocking antibodies and inhibited estrogen-induced Ca^2+^ mobilization.

We found that GPER1 antagonist exacerbates macrophages and Ly6C^high^ monocytes infiltration and inflammatory cytokines secretion in pristane-induced SLE mice, which supported the notion that GPER1 exerts an anti-inflammatory effect in the monocyte/macrophage population.^5^ Monocytes have been increasingly recognized to play a dynamic role in initiating and perpetuating SLE, given their hallmark functions in phagocytosis, antigen presentation, and cytokine production. Our data showed that the anti-GPER1 autoantibodies abrogated the anti-inflammatory capacity of E2, resulting in increased production of IFN-α, TNF-α and IL-6 in monocytes, which are known to be associated with SLE development and tissue damage.

In conclusion, our findings suggest that anti-GPER1 autoantibodies might be novel biomarkers for SLE diagnosis and activity evaluation. Additionally, anti-GPER1 autoantibodies act as blocking antibodies and reverse the inhibitory effects of E2 on inflammatory cytokines secretion through GPER1. Consequently, these results may contribute to understanding the complex pathogenic mechanisms underlying SLE. Therefore, expanding the knowledge of the pathophysiological roles of autoantibodies against estrogen receptors will open avenues for new therapeutic approaches.

## Supplementary information

Supplementary materials

## Data Availability

All relevant data are available in Supplementary Information and from the authors.
