# Supplementary material for: Autoantibodies targeting to GPER1 promote monocyte cytokines production and inflammation in systemic lupus erythematosus
Source: Signal Transduct Target Ther. 2023 Mar 3;8:93. doi: 10.1038/s41392-022-01294-3 (PMC9981603; doi:10.1038/s41392-022-01294-3)

Supplementary Materials for

Autoantibodies Targeting to GPER1 Promote Monocyte Cytokines Production and Inflammation in Systemic Lupus Erythematosus

Xinwei Zhang, Hongyan Qian, Yangchun Chen, Yuanhui Wu, Yuechi Sun, Yan He, Shiju Chen, Yuan Liu and Guixiu Shi

Correspondence to: Guixiu Shi (gshi@xmu.edu.cn) or Yuan Liu (liuyuan@xmu.edu.cn)

**This PDF file includes:**

Materials and Methods

Figure S1 to S3

Tables S1 to S2

Original Film of Western Blot

Materials and Methods

Patients and healthy controls:

We analyzed the sera from 117 consecutive patients with SLE. These patients comprised 104 women and 13 men with a median age of 36 (14 to 69). All patients were diagnosed according to the American College of Rheumatology (ACR) revised criteria. All clinical data of SLE patients were tested and provided by First Affiliated Hospital of Xiamen University. Disease activity categories were defined based on the SLEDAI scores. Patients with other autoimmune diseases, such as rheumatoid arthritis (RA), Sjögren's syndrome (SS), Ankylosing Spondylitis (AS), and gout were also enrolled in this study. 70 healthy volunteers without autoimmune diseases or cancer and matched for age and sex with the SLE group were chosen as healthy controls. All serum samples were obtained by standard methods and stored at -78 °C until used. All patients and healthy volunteers gave written informed consent before participating in this study. The study was reviewed and approved by the Local Ethical Committee of the First Affiliated Hospital of Xiamen University.

Enzyme-linked immunosorbent assay (ELISA)

We analyzed serum IgG immunoreactivity to GPER1 by ELISA in healthy donors, patients with SLE and other autoimmune diseases. Briefly, polystyrene plates (Thermo, USA) were coated with recombinant GPER1 protein (Abnova, 200 ng/well) and incubated overnight at 4°C. The plates were blocked with 5% BSA buffer for 1 hour at room temperate. Serum samples were diluted at 1:200 in sample buffer and incubated for another 1 hour at room temperature. Peroxidase-conjugated rabbit anti-human IgG (Cell Signaling Technology, USA) was used as the secondary antibody, and the substrate 3,3’, 5,5’-tetramethylbenzidine (TMB)/hydrogen peroxide (H_2_O_2_) (RD system, USA) was used as the detecting reagent. The optical density (OD) values were measured at 450 and 620 nm and then used for data analysis. Each serum sample was tested in duplicate. We designated the positive cutoff as the mean OD of the 70 healthy control serum samples + 2 standard deviations (SDs). Human cytokine ELISA kit IL-6 (9.4-600 pg/mL), human cytokine ELISA kit IL-1β (3.9-250 pg/mL), human cytokine ELISA kit TNF-α (15.6-1000 pg/mL), mouse cytokine ELISA kit IL-6 (15.6-1000 pg/mL), and mouse cytokine ELISA kit TNF-α (31.2-2000 pg/mL) were purchased from R&D Systems. Mouse cytokine ELISA kit IFN-α (2.38-152 pg/mL) was purchased from PBL ASSAY SCIENCE. Human cytokine ELISA kit IFN-α (11-700 pg/mL) was purchased from NeoBioscience.

Western blotting

Represented serum samples positive for anti-GPER1 as determined by ELISA were further confirmed by western blotting. The serum samples comprised the 10 patients with the highest OD values and the 5 randomly selected healthy controls. Recombinant GPER1 protein was separated by 10% SDS-PAGE and transferred to a nitrocellulose membrane (Thermo, USA). Then, the nitrocellulose membrane was cut into strips and blocked with PBS containing 5% BSA and 0.2% Tween-20 for 1 hour at room temperature. The nitrocellulose strips were incubated with serum samples at 1:200 dilution for 2 hours at room temperature. Finally, the strips were incubated with peroxidase-conjugated rabbit anti-human IgG for 1 hour at room temperature. The graphs reflect the chemiluminescence (BioRad, USA) values calculated according to the manufacturer’s instructions.

Purification of specific autoantibodies from patient sera

Recombinant GPER1 protein (50 μg; Abnova) was spotted on a nitrocellulose filter and incubated with sera from 10 SLE patients that had OD ＞1 by ELISA for anti-GPER1 antibodies purification. After washing with PBST, the antibodies were eluted with 100 mM glycine, pH 2.5, and mixed for 10 minutes. The eluted antibodies were immediately neutralized with 1M Tris HCl, pH 8, and dialyzed against PBS. Antibodies from sera of healthy donors were used as control.

Transfection and calcium flux assay

HEK293 cells were transfected with a GPER1 construct, consisting of the full-length cDNA ligated into the expression vector p3×flag-CMV-10, using jet PRIME Reagent (Polyplus transfection). 48 hours later, HEK293 cells were pretreated with 10 µg/mL purified α-GPER1 autoantibodies (α-GPER1 Abs) or IgG from healthy donors (HC IgG) for 1 hour. The cells were loaded with 1mM Fluo-4 AM (Thermo Fisher Scientific), washed twice with PBS, and stimulated by 100 nM 17β-estradiol (Sigma). Calcium flux changes were recorded using a Leica SP5 confocal laser-scanning microscope (Leica Microsystems, Wetzlar, Germany). Images were acquired every 1 s and the first 18 s were used as the basal calcium flux line before any stimulation was applied. Calcium flux intensity was analyzed using Leica System Analysis Software.

Cell culture and treatments for functional studies

Peripheral blood mononuclear cells (PBMCs) were isolated by Ficoll-Hypaque density gradient centrifugation (Thermo, USA) according to the manufacturer’s protocol. Cells were cultured in RPMI 1640 medium without phenol red with 10% fetal bovine serum. Cells were treated with 0.01 µg/mL LPS, 100 nM 17β-estradiol, 10 µg/mL purified α-GPER1 Abs or HC IgG for 4 hours. Cells were incubated at 37 °C before cytokine detection by intracellular staining. Separation of monocytes from PBMCs was performed by immunomagnetic-based depletion of non-monocytes using the Pan Monocyte Isolation Kit (Miltenyi Biotec). Cells were treated as above for 72 hours, the supernatants were collected, and IL-1β, IL-6, TNF-α, and IFN-α were measured by ELISA (R&D system, USA).

Pristane-induced lupus mouse model

For lupus-like disease induction, C57BL/6 mice were administered one i.p. injection of 500 μL saline (as a control) or pristane (Sigma). After 2 days, mice were i.p. injected with G15 or DMSO as control every 2 days for 4 weeks. Experimental mice were sacrificed, and the peritoneal lavage fluid was collected for cell counts and cytokine levels.

Flow cytometry

Fc receptors were blocked with Fc block and the dead cells were detected using Fixable Viability Aqua Fluorescent Reactive Dye (Invitrogen) before cell surface staining. Data were acquired using Cytoflex (Beckman Coulter) and analyzed using FlowJo software (Tree Star). The following antibodies were used: FITC anti–human CD14 (Invitrogen), FITC anti–human CD3 (Biolengend), PE anti–human CD14 (Biolengend), Percp/Cy5.5 anti-human CD11c (Biolengend), PE/Cy7 anti–human CD19 (Biolengend), PE/Cy7 anti-human CD56 (Biolengend), PE/Cy7 anti-human HLA-DR (Biolengend), APC anti–human TNF-α (Invitrogen), eFlour450 anti–human IL-6 (Invitrogen), APC anti–human CD80 (Biolengend), APC anti–human TLR4 (Biolengend), anti-human GPER1 (R&D), APC anti-Goat-IgG (R&D).

Statistical analysis

All data are presented as the mean ± SEM. Unpaired T-test, one-way ANOVA, Chi-square test and Spearman’s rank correlation analyses were performed using SPSS19.0 software, and p<0.05 was considered statistically significant.


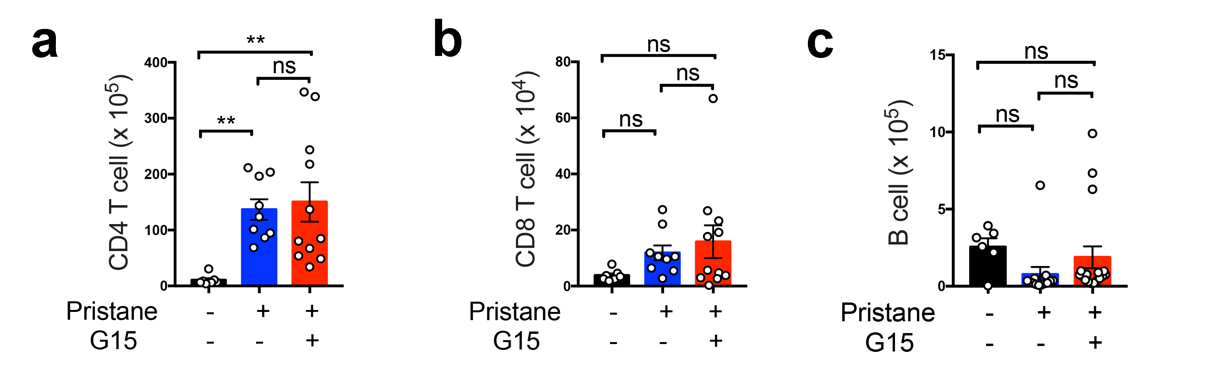


Figure. S1. The numbers of infiltrating T cells and B cells in pristane-induced SLE mice were not affected by G15. (a-c) C57BL/6 mice were treated with or without pristane. After 2 days, mice were i.p. injected with G15 or DMSO as control every 2 days for 4 weeks. Subpopulations of peritoneal cells were identified by flow cytometry as CD4 T cells (CD4^+^), CD8 T cells (CD8^+^), and B cells (CD19^+^). Shown is the total number of each population. Data are represented as mean ± SEM. One-way ANOVA was used to analyze data for differences. ***P* < 0.01.


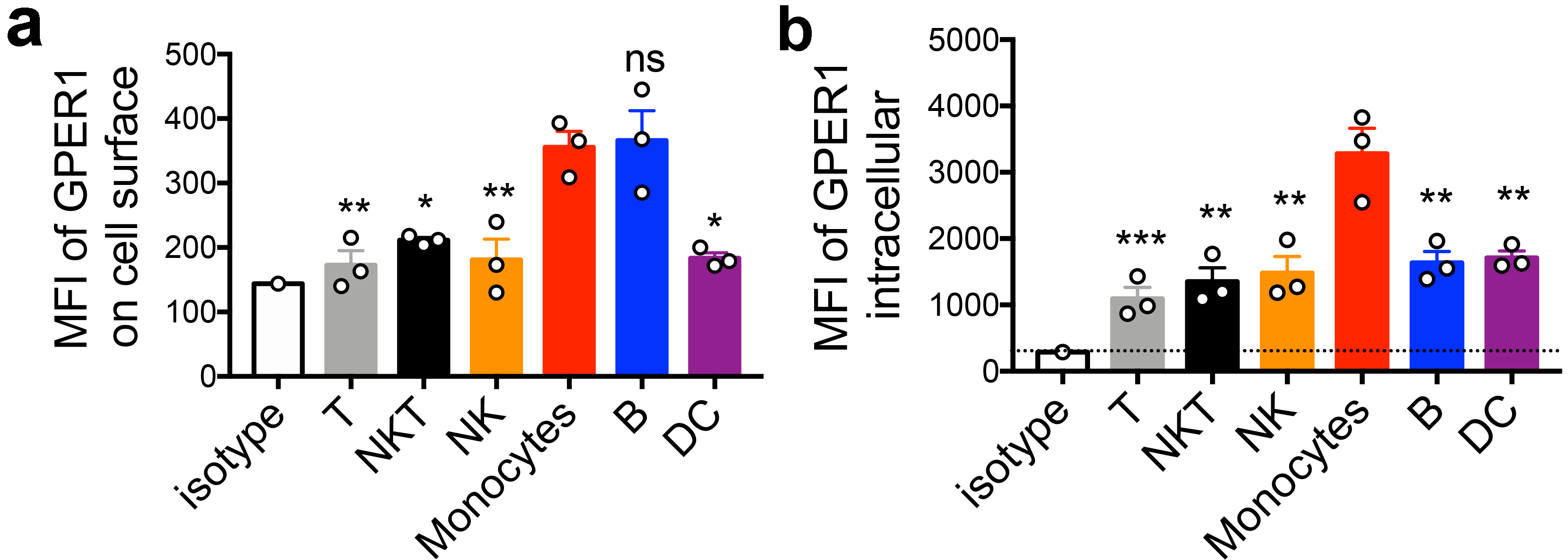


Figure. S2. The expression of GPER1 on the cell surface and intracellular in PBMC subpopulations. (a) Flow cytometry analysis of GPER1 expressions in T cells (CD3^+^CD56^-^), NKT cells (CD3^+^CD56^+^), NK cells (CD3^-^CD56^+^), monocytes (CD14^+^CD3^-^CD19^-^), B cells (CD19^+^), and DC (CD11c^+^CD14^-^) in PBMCs obtained from healthy donor. (b) Intracellular staining of GPER1 in PBMC subpopulations after fixation and permeabilization. Data are represented as mean ± SEM. One-way ANOVA was used to analyze data for differences, * as compared to monocytes. **P* <0.05, ***P* < 0.01, ****P* < 0.001.


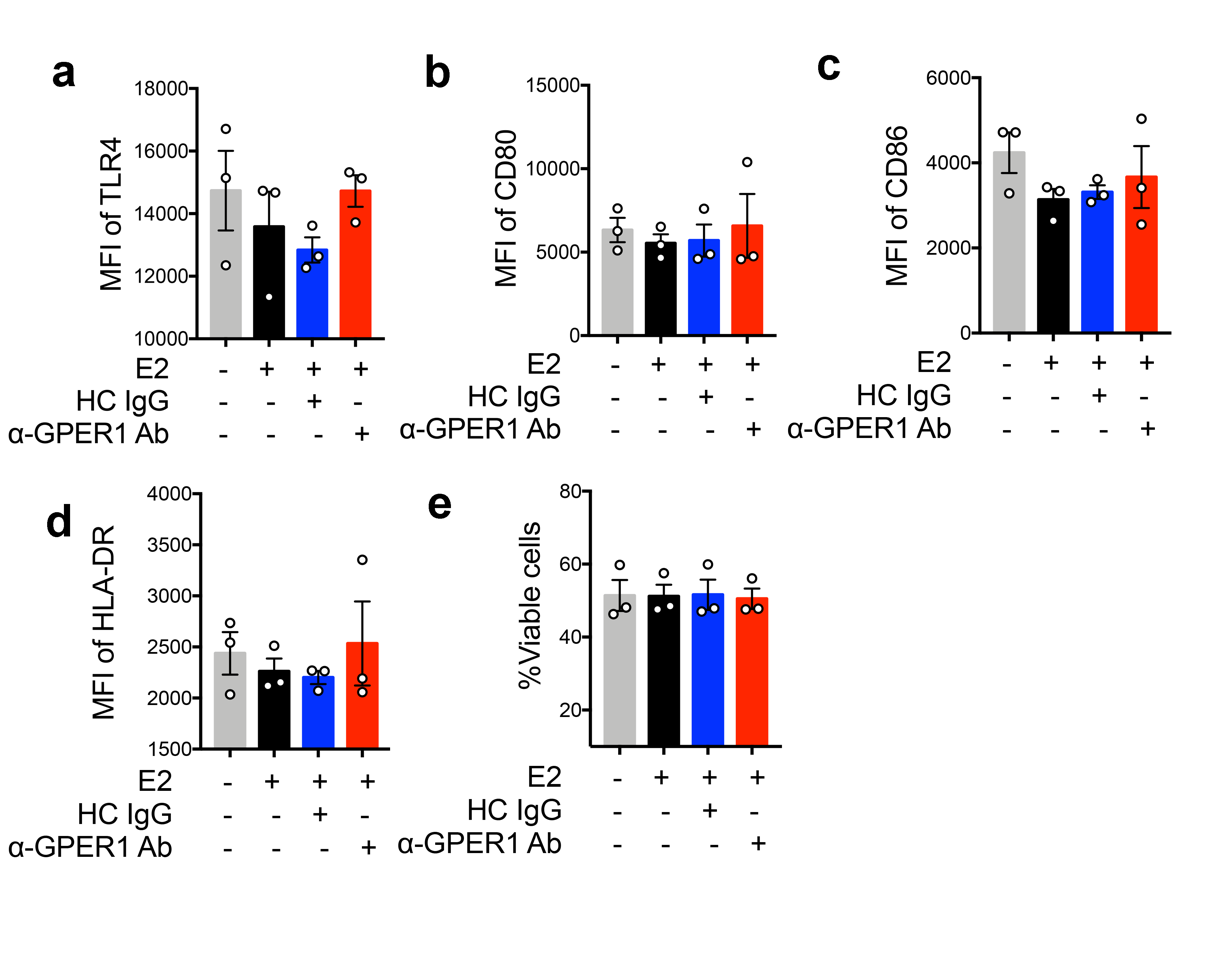


Figure. S3. The expression of TLR4, CD80, CD86, and HLA-DR on monocytes. (a-d) Monocytes were isolated from PBMCs by magnetic cell sorting and were stimulated with α-GPER1 Abs with the presence of E2 for 72 hours. Flow cytometry analysis of TLR4, CD80, CD86, and HLA-DR expressions. (e) Cell viability was studied using flow cytometry. Data are represented as mean ± SEM. One-way ANOVA was used to analyze data for differences.

Table S1. Prevalence of anti-GPER1 autoantibodies in patients with autoimmune diseases and healthy controls.

|  | Number | Age, median (range) years | Anti-GPER1 IgG-positive | Anti-GPER1 IgG-negative | | Frequency |
| --- | --- | --- | --- | --- | --- | --- |
| SLE | 117 | 36 (14-69) | 46 | 71 | 39.3% *** | |
| RA | 56 | 58 (33-81) | 2 | 54 | 3.5% | |
| SS | 60 | 49 (16-69) | 5 | 55 | 8.3% | |
| AS | 61 | 33 (22-66) | 4 | 57 | 6.6% | |
| Gout | 66 | 55 (31-83) | 6 | 60 | 9.1% | |
| Healthy controls | 70 | 32 (24-38) | 4 | 66 | 5.7% | |

***, *P* < 0.001, the chi-square test was used.

Table S2. Clinical characteristics of the 86 SLE patients according to the presence or absence of serum anti-GPER1 antibodies

|  | Anti-GPER1 IgG-positive patients (n=37) | Anti-GPER1 IgG-negative patients (n=49) | p value |
| --- | --- | --- | --- |
| Arthritis | 6 (16.2 %) | 3 (6.12%) | 0.13 |
| Blood system involvement | 11 (29.7 %) | 11 (22.4 %) | 0.44 |
| Kidney involvement | 13 (35.1 %) | 14 (28.6 %) | 0.52 |
| Skin lesions | 12 (32.4 %) | 13 (26.5 %) | 0.55 |
| Low complement | 30 (81.1 %) | 30 (61.2 %) | 0.0471* |
| Fever | 3 (8.1 %) | 2 (4.1 %) | 0.43 |
| dsDNA antibody | 20 (54.1 %) | 16 (32.7%) | 0.0464* |
| Serositis | 15 (40.5%) | 4 (8.2%) | 0.0003 *** |
| Vasculitis | 1 (2.7%) | 1 (2.0%) | 0.84 |
| Oral ulcer | 4 (10.8 %) | 4 (8.2%) | 0.68 |

Date was presented as n (%), **, *P* < 0.01, the chi-square test was used.

Original Film of Western Blot


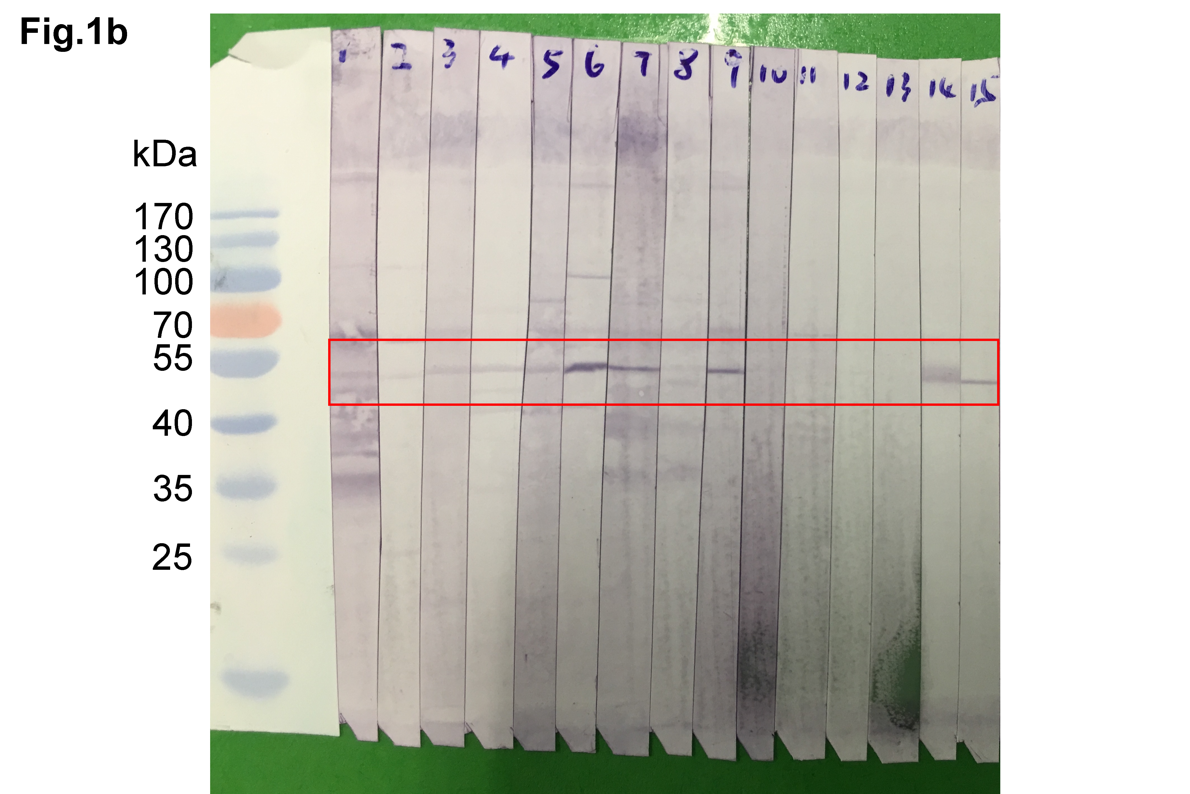

Supplement: Supplementary file 1 — Supplementary materials [file 41392_2022_1294_MOESM1_ESM.docx]
